# Retraction Note: Unraveling the influence of TiO_2_ nanoparticles on growth, physiological and phytochemical characteristics of *Mentha piperita* L. in cadmium-contaminated soil

**DOI:** 10.1038/s41598-025-05650-5

**Published:** 2025-06-23

**Authors:** Hamid Mohammadi, Zahra Kazemi, Ahmad Aghaee, Saeid Hazrati, Rosa Golzari Dehno, Mansour Ghorbanpour

**Affiliations:** 1 Faculty of Agriculture, Azarbaijan Shahid Madani University, Tabriz, Iran; 2 Department of Biology, Faculty of Science, University of Maragheh, Maragheh, Iran; 3 Department of Agriculture, Chalus Branch, Islamic Azad University, Chalus, Iran; 4 Department of Medicinal Plants, Faculty of Agriculture and Natural Resources, Arak University, Arak, 38156-8-8349 Iran; 5 Institute of Nanoscience and Nanotechnology, Arak University, Arak, 38156-8-8349 Iran

Retraction of: *Scientific Reports* 10.1038/s41598-023-49666-1, published online 14 December 2023

The Editors have retracted this Article.

After publication, concerns were raised regarding the data presented in this Article.

• Figure 2A appears to have been inappropriately manipulated and to contain a repeat feature overlayed on the image.

• Figure 1A of this Article appears to be similar to Fig. 1 in^1^ and Fig. 1 in^2^ representing a different material.

Authors were able to provide the data underlying Fig. 2A, but the image does not match the published version. The Editors therefore no longer have confidence in the research presented in this work.

Mansour Ghorbanpour did not explicitly state whether they agree or disagree with the retraction. Hamid Mohammadi, Zahra Kazemi, Ahmad Aghaee, Saeid Hazrati, and Rosa Golzari Dehno did not respond to the Editors correspondence about this retraction.
